# Steroids Versus Immunomodulators in Cardiac Sarcoidosis: A Systematic Review

**DOI:** 10.7759/cureus.80461

**Published:** 2025-03-12

**Authors:** Moiuz Chaudhri, Brandon Goodwin, Raviv Markovitz, Hanna Brancaccio, Mohamad Hammad, Frederick Acquah, Arthur Okere

**Affiliations:** 1 Internal Medicine, Hackensack Meridian Health Ocean University Medical Center, Brick, USA; 2 Internal Medicine, Rowan-Virtua School of Osteopathic Medicine, Stratford, USA

**Keywords:** cardiac sarcoidosis, comparative analysis, corticosteroids, disease management, granulomatous myocarditis, immunomodulators, immunosuppressive therapy, steroids, systematic review, treatment outcomes

## Abstract

Granulomatous inflammation of the heart causes arrhythmias, heart block, and heart failure in cardiac sarcoidosis (CS), a rare but potentially deadly condition. There is increasing interest in using immunomodulators as steroid-sparing medicines, even though corticosteroids are still the usual treatment. This study compared corticosteroids and immunomodulatory treatments through a systematic review and meta-analysis. After a thorough literature search in October 2024, 11 pertinent studies were found. These included observational studies, case series, and randomized controlled trials. Based on changes in myocardial inflammation (SUVmax) and left ventricular ejection fraction (LVEF), the effectiveness of corticosteroids, methotrexate, infliximab, rituximab, and their combinations was evaluated. The analysis revealed that all treatments significantly reduced myocardial inflammation, with methotrexate showing the highest effect size (d = 1.65, p < 0.001). Infliximab in combination with corticosteroids also demonstrated a significant reduction in SUVmax (d = 1.61, p < 0.001). LVEF improved across all treatment groups, although the effect was modest, with infliximab and corticosteroids showing the most significant increase in LVEF (d = 0.4, p = 0.05). The differences between subgroups were not statistically significant (p = 0.46 for SUVmax, p = 0.36 for LVEF). Corticosteroids remain the standard first-line treatment for CS, while methotrexate has shown the highest effect size for reducing myocardial inflammation, supporting its role as a steroid-sparing agent with fewer long-term side effects. Infliximab is effective but carries risk of infection. These findings highlight the need for customized treatment regimens in the management of CS. This study underscores the urgent need for more research to enhance combination medications, pinpoint patient subgroups that may benefit most from specific therapies, and enhance treatment regimens in the field of CS.

## Introduction and background

Sarcoidosis is a multisystem inflammatory disease that is brought on by a confluence of immunological, genetic, and environmental variables. Noncaseating granulomas, which are found primarily in the skin, lungs, eyes, lymph nodes, and heart, are its defining feature. A rare but potentially deadly condition is cardiac sarcoidosis (CS), which involves granulomatous inflammation of the heart [1,2]. Worldwide, there are 10-60 cases of sarcoidosis for every 100,000 people, whereas CS is identified much less frequently, most likely as a result of underdiagnosis. Compared to Northern Europeans, African Americans are more likely to develop sarcoidosis, which can cause more serious symptoms, such as involvement of the heart. Although postmortem investigations indicate subclinical cardiac involvement in 25-70% of cases, clinically detectable cardiac involvement is found in 5-10% of sarcoidosis patients in Western countries [1,3,4]. With 25-30% of sarcoidosis patients presenting with cardiac involvement, a significant contributing factor to sarcoidosis-related mortality, Japan reports a greater prevalence of CS [5].

CS, which generally appears in people between the ages of 25 and 45, is a condition that demands urgent attention. It primarily affects men, while systemic sarcoidosis is more common in women [4]. There are noticeable ethnic variances, with African American and Japanese populations being more prone to severe heart disease. Severe systemic disease and extrapulmonary sarcoidosis affecting the skin, eyes, or nervous system are risk factors for cardiac involvement. A family history of sarcoidosis may raise the risk, even if the genetic predisposition to CS is not well understood. Particularly in patients with significant myocardial involvement, CS raises morbidity and mortality from arrhythmias, heart block, and heart failure. Sudden cardiac death, a significant consequence of CS, underscores the urgency and seriousness of this condition [2,4]. In individuals with sarcoidosis, the CS mortality rate varies from 1% to 7%; however, more excellent rates are noted in Japan [5].

T-helper cells and pro-inflammatory cytokines such as TNF-α, IL-2, and IFN-γ play crucial roles in the pathophysiology of CS [6]. Granuloma formation in CS is driven by an exaggerated T-helper (Th1) immune response, characterized by elevated levels of TNF-α, IL-2, and IFN-γ. These cytokines promote macrophage activation, leading to the persistence of granulomatous inflammation [6]. Corticosteroids suppress this inflammatory cascade by broadly inhibiting cytokine production. Immunomodulators such as methotrexate and azathioprine act by reducing T-cell proliferation and cytokine release, while TNF-α inhibitors (e.g., infliximab) specifically block TNF-α signaling, a key driver of granuloma persistence [6]. These cells and cytokines are involved in the granulomatous infiltration of the heart and conduction system. Myocardial fibrosis, a consequence of chronic inflammation, damages the heart and increases the risk of arrhythmias, heart block, and heart failure. Granulomatous infiltration causes ventricular arrhythmias, such as ventricular tachycardia or fibrillation, which may lead to sudden cardiac death. On the other hand, fibrosis in the conduction system can produce heart block to variable degrees. Although the illness frequently mimics other cardiomyopathies, making diagnosis more complex, advanced imaging techniques like cardiac MRI and PET scans have improved detection [6].

About 25% of those with systemic sarcoidosis have CS, whereas another 25% may have localized cardiac involvement [2]. Left ventricular ejection fraction (LVEF) is a key predictor of prognosis; lower levels are associated with worse outcomes [6,7]. The first-line treatment for arrhythmias and inflammation reduction is corticosteroids, especially prednisone; however, prolonged use of these medications carries hazards, including infections, osteoporosis, and hyperglycemia [4,7-9]. To reduce side effects and enhance disease management, immunomodulators such as methotrexate, azathioprine, and mycophenolate mofetil are being utilized more and more as steroid-sparing medications [10]. Infliximab and other tumor necrosis factor-alpha inhibitors work well in refractory patients, but they have drawbacks, such as the potential for latent TB reactivation [11,12]. Methotrexate, azathioprine, and mycophenolate mofetil were chosen for their established steroid-sparing activity in sarcoidosis (according to retrospective and observational studies that demonstrated efficacy in maintaining disease control). Methotrexate is the most widely used and best-studied agent for use in CS [10]. Infliximab is being used on the basis of its strong TNF-α inhibition effect proved to be effective in refractory sarcoidosis [11]. These agents constitute the most studied immunomodulators to date in CS and therefore have been chosen for comparison in this meta-analysis. Although corticosteroids are widely employed, the ideal dosing duration and tapering strategy in CS have not been established. Moreover, whereas immunomodulators have an important role as steroid-sparing agents, their relative efficacy, optimal sequencing, and long-term safety profile await further study. Likewise, there is insufficient data to direct patient selection for TNF-α inhibitors, as they may be beneficial but also have significant infection risk. These gaps highlight the importance of rigorous comparative studies to guide evidence-based treatment algorithms. The need for individualized therapy is paramount in the optimal management of CS, as it can reduce treatment-related side effects, prevent problems, and regulate inflammation, thereby emphasizing the importance of personalized care.

## Review

Rationale for the systematic review

Immunomodulators have been increasingly investigated as steroid-sparing agents, yet their relative therapeutic effectiveness is unclear. In the absence of a consensus on the best treatment strategy, a systematic comparison of corticosteroids with immunomodulators is essential to facilitate clinical decision-making and mitigate the global burden of steroid-related complications.

Methods

A systematic review and comparative analysis were conducted utilizing the Preferred Reporting Items for Systematic reviews and Meta-Analyses (PRISMA) 2020 guidelines. This review sought to compare the efficacy of corticosteroids versus immunomodulators in patients afflicted with CS. This review was registered online ahead of study conduction through the University of York’s PROSPERO system (registration number CRD42024596710).

Inclusion Criteria

For an article to be included in the analysis, it must contain primary data on the use of either corticosteroids or immunomodulators. Studies must include patients who have been diagnosed with CS, regardless of the duration of time from the initial diagnosis. Specific types of studies to be included in the analysis are randomized controlled trials (RCTs), cohort studies, retrospective studies, and case series containing more than one patient. For articles to be included, they must contain adequately reported baseline and post-treatment data to be used in statistical testing. Studies that do not provide adequate data or unclear data will have their corresponding author contacted for clarification, and a period of four weeks will be given to allow for a response. If there is no response, the study is to be excluded. Patients must be over the age of 18 years. Studies must report on LVEF or SUVmax, as they are our outcomes of interest. Articles were not excluded based on country of origin, date, or original language.

Exclusion Criteria

Articles are excluded if they do not contain an interventional group of either immunomodulators or corticosteroids. If patients were suspected of having sarcoidosis but not officially diagnosed, they were excluded. Articles that do not have accessible full-texts despite exhaustive searching, contacting the corresponding author, or utilization of the interlibrary loan system were excluded. Study types excluded are single-patient case reports, gray literature, conference abstracts or posters, systematic reviews, or other review article types not containing primary data. Articles with mixed or inadequately reported data were excluded. Articles that did not investigate LVEF or SUVmax were also excluded.

Information Sources and Search Strategy

This systematic review and comparative analysis used four databases for article retrieval: PubMed, Embase, Cochrane Libraries, and Web of Science. Initial article retrieval and database querying occurred on 10/12/2024. A single search string was used between all four databases to ensure accuracy and continuity between the four databases. The string was initially formed by identifying MeSH terms for the variables of interest and the subsequent addition of commonly known alternative names. Boolean operators were then added to the string. The search string used was: (“corticosteroids” OR “corticosteroid” OR “glucocorticoids” OR “glucocorticoids” OR “immunosuppressant” OR “immunosuppressants” OR “immunosuppressive agent” OR “immunosuppressive agents” OR “immunomodulators” OR “immunomodulator”) AND (“cardiac sarcoidosis” OR “cardiac sarcoid”). To ensure accurate retrieval of all relevant articles, manual searching of the databases was also conducted to look for cited-by and similar-to articles.

Articles were uploaded from EndNote to Rayyan.ai and duplicate detection was conducted with two independent reviewers manually searching through the remaining articles to ensure accuracy and misses. Database-specific adjustments were not required.

Study Selection

Following duplicate detection, articles were thoroughly appraised by their titles and abstracts to determine if they met inclusion and exclusion criteria. After this, the remaining articles were meticulously appraised for full-text relevance.

**Figure 1:**
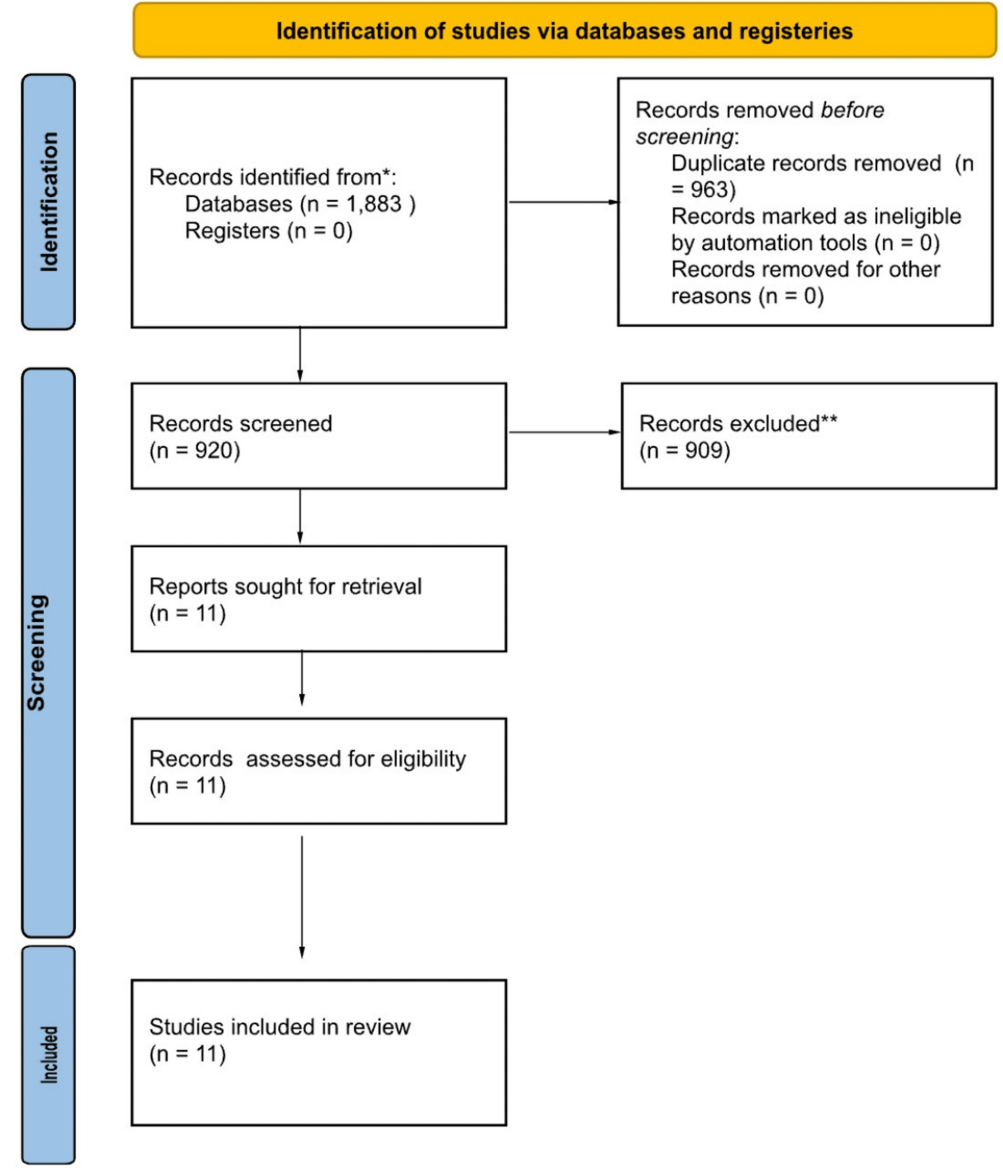
PRISMA flow diagram of study selection PRISMA, Preferred Reporting Items for Systematic reviews and Meta-Analyses

Article screening was conducted independently by (MC) and (BG), with a third (NP) being brought in to adjudicate any ties. In addition, the third screener checked for consistency and bias in the screening process. Following this stepwise process, articles that satisfied all inclusion and exclusion criteria were appraised for relevant data. Articles without full text would have their corresponding author contacted, a total of three attempts, and a period of four weeks was given for them to respond. If the author did not respond, the article was excluded at this step. The stepwise process of article selection can be seen in Figure 1.

Data Collection

Relevant articles that contained adequately reported statistical data were included and their data was extracted into a separate data extraction sheet. Outcomes of interest extracted into the spreadsheet are LVEF and SUVmax. Patient demographics, time from initial diagnosis, dose of treatment, and study duration were also extracted. The relevant extracted data was then uploaded to the IBM SPSS Statistics for Windows, Version 21.0 (Released 2012; IBM Corp., Armonk, NY, USA) for comparative analysis [13].

Certainty of Evidence and Risk of Bias Assessment

Included articles were also then appraised for quality and certainty of evidence using the Grading of Recommendations Assessment, Development and Evaluation (GRADE) criteria [14]. The subsequent results of GRADE analysis and explanations of whether data quality and strength were upgraded or downgraded can be visualized in the summary chart (adapted from GRADEpro GDT) below.

The remaining articles were also appraised for potential bias based on specific metrics for their respective study design. For instance, ROBINS-I would be employed to search for bias in RCTs and observational studies. A description of the determinations of ROBINS-I analysis can be found below (derived from Robvis).

Results

An initial article search and database query were conducted on 10/12/2024. This search resulted in a total of 1,883 articles. Of these 1,883 articles, 963 were duplicates and removed after AI article duplicate detection and manual article-duplicate double-checking. The remaining articles were then screened for title and abstract relevance, leaving 11 articles for full-text screening (Table 1). These 11 remaining articles were deemed to be relevant and have pertinent data and were subsequently extracted and used in analysis. Of these remaining studies, two were RCTs, eight were observational (both retrospective and prospective), and one was a case series (Table 1).

**Table 1:** Studies included in this review

Author (year)	Study origin country	Title	Reference number
Chiu et al. (2005)	Japan	Prevention of left ventricular remodeling by long-term corticosteroid therapy in patients with cardiac sarcoidosis	[15]
Griffin et al. (2021)	United States	Management of cardiac sarcoidosis using mycophenolate mofetil as a steroid-sparing agent	[16]
Bakker et al. (2021)	Netherlands	Effectiveness and safety of infliximab in cardiac sarcoidosis	[17]
Kato et al. (2003)	Japan	Efficacy of corticosteroids in sarcoidosis presenting with atrioventricular block	[18]
Vis et al. (2023)	Netherlands	Prednisone vs methotrexate in treatment naïve cardiac sarcoidosis	[19]
Nagai et al. (2014)	Japan	Treatment with methotrexate and low-dose corticosteroids in sarcoidosis patients with cardiac lesions	[20]
Harper et al. (2019)	United States	Infliximab for refractory cardiac sarcoidosis	[21]
Suwa et al. (2024)	Japan	Cardiac sarcoidosis treated with nonsteroidal immunosuppressive therapy	[22]
Morimoto et al. (2024)	Japan	Prospective analysis of immunosuppressive therapy in cardiac sarcoidosis with fluorodeoxyglucose myocardial accumulation: the PRESTIGE study	[23]
Elwazir et al. (2022)	United States	Rituximab for the treatment of refractory cardiac sarcoidosis: a single-center experience	[24]
Wand et al. (2022)	United States	Effect of corticosteroids on left ventricular function in patients with cardiac sarcoidosis	[25]

Effect of Intervention

Initial heterogeneity testing denoted a result of I² = 15%, which is under the threshold set by Cochrane to denote a small degree of heterogeneity. As such, fixed-effect models were employed for statistical comparative analysis. 

The change in SUVmax between all groups denoted an overall decrease, with a pooled overall effect size of 1.41 (p < 0.001; 95% CI: 0.93-1.88). Between the groups investigated, infliximab and standard of care (corticosteroid) denoted an effect size of 1.61 (p < 0.001; 95% CI: 0.93-2.29), rituximab plus corticosteroids denoted an effect size of 0.64 (p = 0.24; 95% CI: 0.43-1.71), corticosteroids only denoted an effect size of 1.19 (p < 0.001; 95% CI: 0.58-1.79), methotrexate only showed an effect size of 1.65 (p < 0.001; 95% CI: 1.11-2.19), and Methotrexate plus corticosteroids denoted an effect size of 1.40 (p < 0.001; 95% CI: 0.93-1.88). However, differences between subgroups were determined not to be statistically significantly different (p = 0.46).

The change in LVEF between all groups depicted a significant overall increase in LVEF. However, there is no significant overall effect size (d = 0.14; p = 0.01). Between the groups investigated, infliximab and standard of care (corticosteroid) denoted an effect size of d = 0.4 (p = 0.05), rituximab plus corticosteroids denoted a small effect size of 0.39 (p = 0.48), corticosteroids only denoted no effect size of d = 0.09 (p = 0.11), methotrexate only showed an effect size of d = 0.31 (p = 0.22), and methotrexate plus corticosteroids denoted an effect size of d = 0.34 (p = 0.06). However, differences between subgroups were determined not to be statistically significantly different (p = 0.36).

Certainty of Evidence and Risk of Bias Assessment

GRADE analysis shown in Table 2 and Table 3 revealed an overall moderate certainty of evidence for the effect of corticosteroids versus immunomodulators on parameters of cardiac function [14]. Indirect comparisons lowered the certainty of evidence across the included studies. Other factors that are taken into account for upgrading the quality of evidence (i.e., large effect size, dose-response gradient, and plausible confounders that would have reduced effect size) were not applicable to the outcomes that were studied in this systematic review.

**Table 2:** Summary of findings of GRADE assessment of certainty of evidence for the effect of corticosteroids vs immunomodulators on LVEF in patients with cardiac sarcoidosis GRADE, Grading of Recommendations Assessment, Development and Evaluation; LVEF, left ventricular ejection fraction; RCT, randomized controlled trial

Participants (studies)	Risk of bias	Inconsistency	Indirectness	Imprecision	Other considerations	Overall certainty of evidence
How does the LVEF compare in corticosteroids vs immunomodulators in patients with cardiac sarcoidosis?						
n (RCTs, observational studies, and case series)	Not serious	Not serious	Serious	Not serious	Undetected	Moderate
Explanation	The risk of bias found among included studies was determined to not substantially lower confidence in the results of this systematic review.	-	Comparing the efficacy of corticosteroids and immunomodulators using different protocols among patients with differing baseline characteristics	-	-	-

**Table 3:** Summary of findings of GRADE assessment of certainty of evidence for the effect of corticosteroids vs immunomodulators on SUVmax in patients with cardiac sarcoidosis GRADE, Grading of Recommendations Assessment, Development and Evaluation; RCT, randomized controlled trial

Participants (studies)	Risk of bias	Inconsistency	Indirectness	Imprecision	Other considerations	Overall certainty of evidence
How does the SUVmax compare in corticosteroids vs immunomodulators in patients with cardiac sarcoidosis?						
n (RCTs, observational studies, and case series)	Not serious	Not serious	Serious	Not serious	Undetected	Moderate
Explanation	The risk of bias found among included studies was determined to not substantially lower confidence in the results of this systematic review.	-	Comparing the efficacy of corticosteroids and immunomodulators using different protocols among patients with differing baseline characteristics	-	-	-

**Figure 2:**
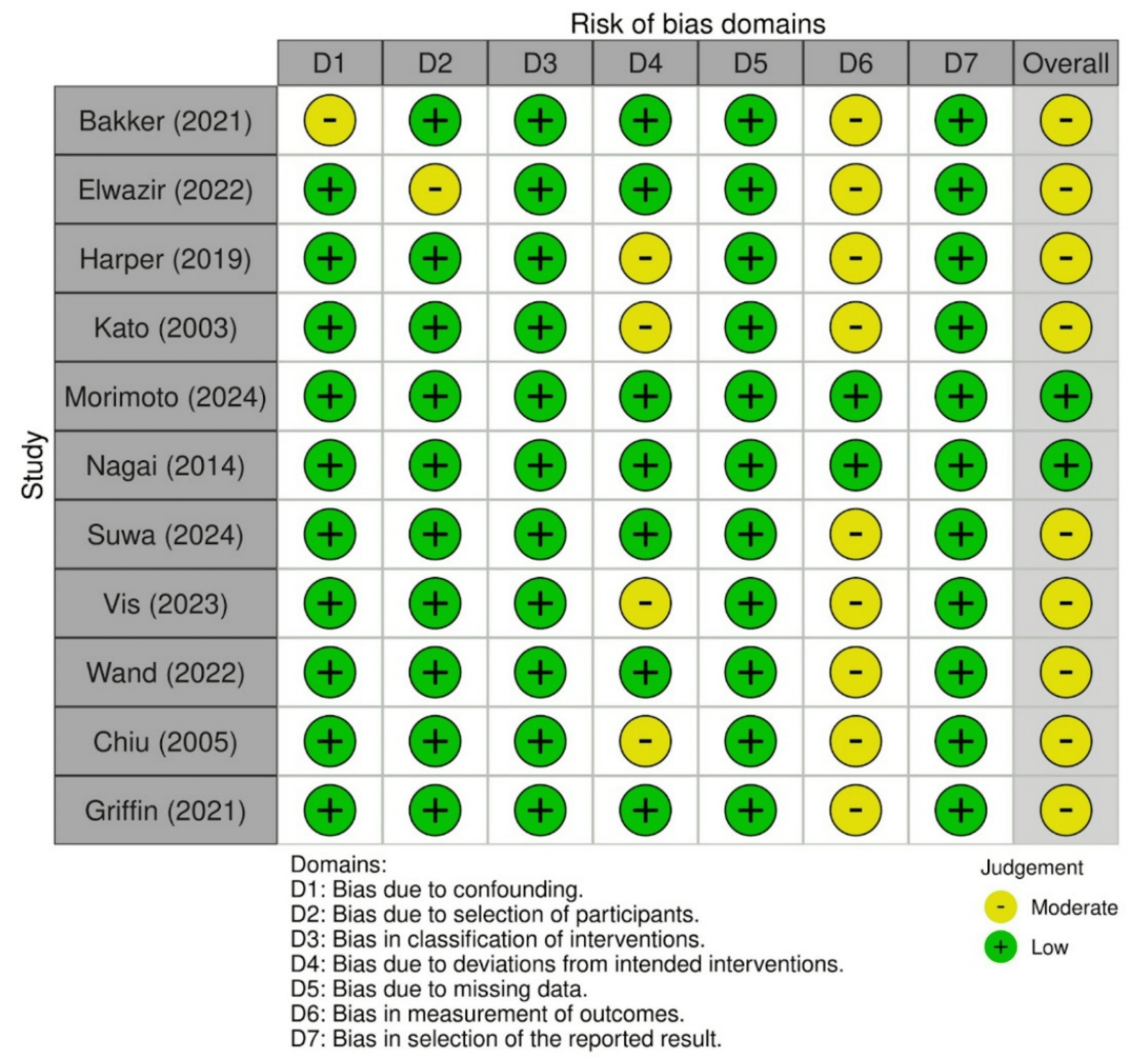
ROBINS-I traffic-light plot Risk of bias assessment using the ROBINS-I tool for included studies. Green indicates low risk, yellow moderate risk, red serious risk, and dark red critical risk of bias. This figure includes data from references [15-25].

**Figure 3:**
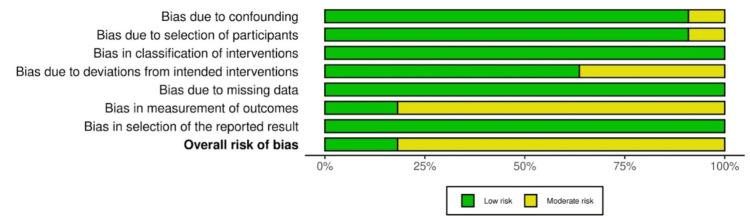
ROBINS-I summary plot

The risk of bias assessment revealed moderate concerns of risk of bias in nine out of the 11 included studies (Figure 2, Figure 3). The concerns for risk of bias arose from confounders [17], selection of participants in the study [24], deviations from the intended intervention [15,18,19,21], and bias in measurement outcome (all nine non-RCTs). Moderate risk of bias in deviations from the intended intervention was determined if there were greater than anticipated occurrences of adverse events that may have influenced the findings of the study; measurement of outcomes was ascertained if outcome assessors were not blinded to the intervention that the participant received. However, the risk of bias present in the studies was not enough to substantially lower confidence in the results of this analysis, hence the decision to score the risk of bias in the GRADE analysis as “not serious.”

Discussion

We present the first and most up-to-date systematic review and comparative analysis of glucocorticoids and immunotherapy for the treatment of CS. Noncaseating granulomas are a hallmark of sarcoidosis, a multisystem inflammatory disease that primarily affects the skin, eyes, lymph nodes, lungs, and heart [5]. Granulomatous inflammation of the cardiac tissues causes CS, a rare but potentially deadly condition that can cause arrhythmias, irregular conduction, heart failure, and sudden cardiac death. Its prevalence varies throughout the world, with higher rates recorded in Japan as a result of the country’s growing usage of sophisticated imaging techniques like cardiac MRI and PET [5]. Systemic sarcoidosis, extrapulmonary involvement, genetic markers, and ethnic predilection are risk factors. Nonspecific symptoms and a lack of conclusive tests make diagnosis difficult. Although long-term use of corticosteroids, especially prednisone, involves significant hazards, immunomodulators such as methotrexate and mycophenolate mofetil are used as steroid-sparing medicines [15-18]. TNF-α inhibitors may be necessary in more advanced cases; however, they carry an infection risk [7]. The degree of the disease affects the prognosis, and one important predictor of results is the LVEF [15].

Our results indicate a significant decrease in SUVmax across all treatment groups, suggesting a widespread decline in myocardial inflammation. Methotrexate alone had the most significant effect size (d = 1.65, p < 0.001), highlighting its potential as a steroid-sparing drug and offering optimism for the future of CS treatment [19,20]. Infliximab plus corticosteroids had the second largest effect size (d = 1.61, p < 0.001). Although infliximab has a higher risk of serious infections, it is a potential treatment for refractory cases [21]. Even though corticosteroids alone also significantly reduced SUVmax (d = 1.19, p < 0.001), their long-term side effects make adjunct medications like methotrexate or infliximab more appealing for long-term treatment [22,23]. Interestingly, the effect size of rituximab with corticosteroids was lower (d = 0.64, p = 0.24), suggesting that this combination may not be as effective as alternative treatment strategies for reducing inflammation. Although SUVmax decreased significantly across groups, there were no statistically significant changes across subgroups (p = 0.46). This could be due to sample size limitations or the diversity of the patient population. These findings add further support to a potential class effect among immunomodulators, as no substantial differences within subgroups were observed, although differences in effect sizes emphasize the need for personalized therapy. Methotrexate achieved the greatest decrease in myocardial inflammation, confirming its status as a steroid-sparing agent; infliximab had the best outcome in terms of LVEF improvement, suggesting that it may be beneficial for refractory cases. Methotrexate reduces TNF-α and IL-6 mechanistically, infliximab blocks TNF-α directly, and rituximab depletes B cells [6]. The smaller effect size of rituximab may suggest a less important role of B-cell-mediated inflammation in CS. These findings imply the early introduction of steroid-sparing agents, methotrexate for stable disease, and infliximab for severe or progressive disease. Studies in the future should further refine the selection of treatment based on biomarkers and risk stratification. Predictors of treatment response and optimization of therapeutic strategies may be specified using a quantitative meta-regression analysis.

LVEF increased significantly across all treatment groups despite the small pooled effect size (d = 0.14, p = 0.01) and nonstatistically significant subgroup differences (p = 0.36). Given its effectiveness in refractory cases, infliximab plus corticosteroids demonstrated the most significant improvement in LVEF (d = 0.4, p = 0.05), which is clinically significant [24,25]. Methotrexate by itself (d = 0.31, p = 0.22) and in conjunction with corticosteroids (d = 0.34, p = 0.06) also showed favorable trends, even if the results did not reach statistical significance. In order to effectively address both inflammation and cardiac function, adjunct medication is required, as corticosteroids alone had no clinically relevant effect size on LVEF (d = 0.09, p = 0.11). The modest improvement in LVEF (d = 0.39, p = 0.48) observed with rituximab plus corticosteroids raises questions about its possible application in the management of CS [17,18,24]. While the results did not reach statistical significance, the trend suggests that this combination may have potential in certain patient subgroups or in combination with other treatments.

Future Directions

The lack of subgroup differences in SUVmax and LVEF suggests that CS is complex, likely in part due to the use of variable imaging techniques and disparate disease activity and response characteristics. Identifying trusted biomarkers - inflammatory markers, PET scan traits, genetic factors, for instance - may help identify which patients will benefit most or least from specific therapies. The incorporation of standardized imaging techniques and outcome measures will also improve our ability to compare results across studies. Although corticosteroids are a first-line treatment, our findings provide evidence that methotrexate is a useful steroid-sparing agent. More data is needed to assess the appropriate dosing strategies and treatment durations to optimize benefits and minimize side effects. Infliximab is effective in refractory cases suggesting that the addition of this drug to methotrexate or other immunomodulators may be a successful approach. Future studies should also investigate whether starting the drugs together or sequentially produces better outcomes.

Limitations

One of the major limitations of this study is the lack of equal representation among all types of interventions. For instance, there was only one study investigating rituximab and corticosteroids on the variables investigated, whereas other variables were more numerous. The underrepresentation of rituximab studies led to wider confidence intervals, limiting the certainty of analyses of subgroups. The risk of bias was moderate overall and particularly for non-RCTs, which also limited the strength of conclusions, as studies not blinded or that lacked standardized assessment of outcomes could have falsely influenced estimates of effect.

Finally, the diversity of geography related to disease presentation and treatment response limits the generalizability of our results. Most studies lacked diverse population data which may make it difficult to determine whether effects vary across the world. Lastly, moderate certainty of evidence was observed due to indirect comparisons in our analysis, suggesting that clinically applicable results would require direct head-to-head trials in the future, which may be difficult.

## Conclusions

Granulomatous inflammation is a hallmark of CS, a rare form of systemic sarcoidosis that can cause serious cardiac problems such as arrhythmias, heart block, and sudden cardiac death. According to this comprehensive analysis, corticosteroids continue to be the most effective treatment for lowering myocardial inflammation, as seen by the notable decreases in SUVmax. The use of immunomodulators such as methotrexate, which showed the most significant effect size in lowering inflammation and appeared as a prospective steroid-sparing drug, is necessary due to the long-term adverse effects of corticosteroids. Although infliximab’s infection concerns necessitate close patient monitoring, it also demonstrated effectiveness in refractory instances. LVEF improved in all treatment groups, although the best outcomes were obtained with combination medications, particularly those with methotrexate and corticosteroids. Subgroup differences did not reach statistical significance, likely due to the complexity and heterogeneity of CS. Understanding the heterogeneity of CS requires that treatment response and disease progression be periodically reassessed in order to guide changes to therapy and optimize long-term outcomes. Further studies are required to address mechanisms behind treatment exerting greater effects on SUVmax compared to LVEF, which have implications for understanding the natural history of the disease and response to therapies. Long-term studies are also necessary to consider the effects of the treatment on survival, quality of life, and long-term treatment effects to further delineate clinical guidelines for patients with CS.

## References

[1] Okada DR, Bravo PE, Vita T (2018). Isolated cardiac sarcoidosis: a focused review of an under-recognized entity. J Nucl Cardiol.

[2] Birnie DH, Nery PB, Ha AC, Beanlands RS (2016). Cardiac sarcoidosis. J Am Coll Cardiol.

[3] Iannuzzi MC, Rybicki BA, Teirstein AS (2007). Sarcoidosis. N Engl J Med.

[4] Yazaki Y, Isobe M, Hiroe M, Sekiguchi M, Central Japan Heart Study Group (2001). Prognostic determinants of long-term survival in Japanese patients with cardiac sarcoidosis treated with prednisone. Am J Cardiol.

[5] Gilotra NA, Griffin JM, Pavlovic N (2022). Sarcoidosis-related cardiomyopathy: current knowledge, challenges, and future perspectives state-of-the-art review. J Card Fail.

[6] Grunewald J, Eklund A (2007). Role of CD4+ T cells in sarcoidosis. Proc Am Thorac Soc.

[7] Trivieri MG, Spagnolo P, Birnie D (2020). Challenges in cardiac and pulmonary sarcoidosis: JACC state-of-the-art review. J Am Coll Cardiol.

[8] Chen ES, Moller DR (2015). Etiologies of sarcoidosis. Clin Rev Allergy Immunol.

[9] Hosoda Y, Sasagawa S, Yasuda N (2002). Epidemiology of sarcoidosis: new frontiers to explore. Curr Opin Pulm Med.

[10] Newman LS, Rose CS, Bresnitz EA (2004). A case control etiologic study of sarcoidosis: environmental and occupational risk factors. Am J Respir Crit Care Med.

[11] Kusano KF, Satomi K (2016). Diagnosis and treatment of cardiac sarcoidosis. Heart.

[12] Kusano KF (2013). Effect of corticosteroid on arrhythmic events in patients with cardiac sarcoidosis. J Cardiol.

[13] IBM Corp. IBM SPSS Statistics. https://www.ibm.com/products/spss-statistics/var?adoper=265413_1&utm_content=SRCWW&p1=Search&p4=43700050715561164&p5=e&p9=58700005519276965&gclsrc=aw.ds&gad_source=1&gclid=CjwKCAjwvr--BhB5EiwAd5YbXkv6YB0mKxUePyTDHsEwuLX2DnxIq-4LC6rV5lbn0RWRnJU_VMch-BoCy_gQAvD_BwE.

[14] (2013). GRADE Handbook for Grading Quality of Evidence and Strength of Recommendations. Updated October.

[15] Chiu CZ, Nakatani S, Zhang G (2005). Prevention of left ventricular remodeling by long-term corticosteroid therapy in patients with cardiac sarcoidosis. Am J Cardiol.

[16] Griffin JM, Chasler J, Wand AL (2021). Management of cardiac sarcoidosis using mycophenolate mofetil as a steroid-sparing agent. J Card Fail.

[17] Bakker AL, Mathijssen H, Azzahhafi J (2021). Effectiveness and safety of infliximab in cardiac sarcoidosis. Int J Cardiol.

[18] Kato Y, Morimoto S, Uemura A, Hiramitsu S, Ito T, Hishida H (2003). Efficacy of corticosteroids in sarcoidosis presenting with atrioventricular block. Sarcoidosis Vasc Diffuse Lung Dis.

[19] Vis R, Mathijssen H, Keijsers RG (2023). Prednisone vs methotrexate in treatment naïve cardiac sarcoidosis. J Nucl Cardiol.

[20] Nagai S, Yokomatsu T, Tanizawa K (2014). Treatment with methotrexate and low-dose corticosteroids in sarcoidosis patients with cardiac lesions. Intern Med.

[21] Harper LJ, McCarthy M, Ribeiro Neto ML (2019). Infliximab for refractory cardiac sarcoidosis. Am J Cardiol.

[22] Suwa K, Naruse Y, Nabeta T (2024). Cardiac sarcoidosis treated with nonsteroidal immunosuppressive therapy. Int J Cardiol Heart Vasc.

[23] Morimoto R, Unno K, Fujita N (2024). Prospective analysis of immunosuppressive therapy in cardiac sarcoidosis with fluorodeoxyglucose myocardial accumulation: the PRESTIGE study. JACC Cardiovasc Imaging.

[24] Elwazir M, Krause ML, Bois JP (2022). Rituximab for the treatment of refractory cardiac sarcoidosis: a single-center experience. J Card Fail.

[25] Wand AL, Pavlovic N, Duvall C (2022). Effect of corticosteroids on left ventricular function in patients with cardiac sarcoidosis. Am J Cardiol.

